# Keratinocyte Function in Normal and Diabetic Wounds and Modulation by FOXO1

**DOI:** 10.1155/2020/3714704

**Published:** 2020-10-28

**Authors:** Yulan Wang, Dana T. Graves

**Affiliations:** ^1^State Key Laboratory Breeding Base of Basic Science of Stomatology (Hubei-MOST) and Key Laboratory of Oral Biomedicine Ministry of Education, School and Hospital of Stomatology, Wuhan University, Wuhan, 430079 Hubei, China; ^2^Department of Periodontics, School of Dental Medicine, University of Pennsylvania, Philadelphia, PA, 19104 Pennsylvania, USA; ^3^Department of Implantology, School and Hospital of Stomatology, Wuhan University, Wuhan, 430079 Hubei, China

## Abstract

Diabetes has a significant and negative impact on wound healing, which involves complex interactions between multiple cell types. Keratinocytes play a crucial role in the healing process by rapidly covering dermal and mucosal wound surfaces to reestablish an epithelial barrier with the outside environment. Keratinocytes produce multiple factors to promote reepithelialization and produce factors that enhance connective tissue repair through the elaboration of mediators that stimulate angiogenesis and production of connective tissue matrix. Among the factors that keratinocytes produce to aid healing are transforming growth factor-*β* (TGF-*β*), vascular endothelial growth factor-A (VEGF-A), connective tissue growth factor (CTGF), and antioxidants. In a diabetic environment, this program is disrupted, and keratinocytes fail to produce growth factors and instead switch to a program that is detrimental to healing. Changes in keratinocyte behavior have been linked to high glucose and advanced glycation end products that alter the activities of the transcription factor, FOXO1. This review examines reepithelialization and factors produced by keratinocytes that upregulate connective tissue healing and angiogenesis and how they are altered by diabetes.

## 1. Reepithelialization

The epidermis covers the dermis, provides a barrier to pathogens, and regulates water release. Approximately 90% of cells in the epidermis are keratinocytes with the remainder consisting of leukocytes such as Langerhans cells, tactile epithelial (Merkel) cells, and melanocytes, which are separated from the underlying dermis by a basement membrane [1]. Barrier function is achieved by adherens junctions formed by cadherins, which are linked to actin filaments in the cytoplasm that link epidermal cells. As a barrier exposed to a complicated environment, the epidermis is primed to repair wounds when disrupted. Reepithelialization, which resurfaces the wound with new epithelium, is an important process in wound healing [2].

Reepithelialization of a skin wound occurs when basal keratinocytes migrate from the wound edge and dermal appendages (hair follicles, sweat glands, and sebaceous glands). Before migration occurs across an open wound, new granulation tissue must form. After initiating epithelial migration, keratinocytes proliferate, providing a sufficient number of cells for subsequent migration. Reepithelialization, thus, requires the formation of a provisional wound bed matrix and the migration and proliferation of keratinocytes.

Keratinocyte migration involves actin filament remodeling of the cytoskeleton and cell attachment and deattachment [2, 3]. For protrusion, actin filaments form lamellipodium that push forward; for traction, they assemble into antiparallel arrays with myosin II [4]. Keratinocyte migration also involves integrins [5] that consist of an alpha and a beta subunit that bind to specific extracellular matrix proteins with relatively high affinity. The integrins *α*6*β*4, *α*3*β*1, and *α*v*β*6 participate in keratinocyte migration. Integrins *α*6*β*4 and *α*3*β*1 bind to laminin, while *α*v*β*6 binds to fibronectin and tenascin [6, 7]. These specific integrin-extracellular matrix protein interactions are critical to reepithelialization since laminin, fibronectin, and tenacin are components of the provisional matrix in the wound bed. Studies examining mice with *β*6 gene deletion or mice treated with anti-*α*v*β*6 antibody exhibit impaired reepithelialization, establishing a mechanistic relationship between their activity and keratinocyte migration in wound healing *in vivo* [8].

Matrix metalloproteinases (MMP) facilitate cell migration by enabling cell–matrix detachment, an essential aspect of movement [9]. The functional importance of MMPs in facilitating keratinocyte migration and wound healing has been shown by use of specific inhibitors and genetic deletion of MMP2 or MMP13. MMP inhibitors or MMP gene deletion delay keratinocyte migration and interfere with reepithelialization *in vivo* [10, 11].

Growth factors that stimulate keratinocyte migration include transforming growth factor-*β* (TGF-*β*), heparin-binding epidermal growth factor-like growth factor (HB-EGF), and fibroblast growth factor (FGF). Lineage-specific TGF-*β*1 deletion in keratinocytes impairs reepithelialization and wound healing [12]. Similarly, inhibition of TGF-*β* activity by antibody treatment results in diminished reepithelialization [13], while application of TGF-*β* can accelerate healing in diabetic animals where it is deficient [14, 15]. Mice with deletion of FGF2 have delays in wound healing linked to reduced keratinocyte migration and impaired lamellipodia formation [16]. A summary of growth factors produced by keratinocytes is provided in Table 1.

## 2. Keratinocyte Migration, Proliferation, and Reepithelialization

Reepithelialization relies on the migration and proliferation of keratinocytes [17]. Migration of keratinocytes occurs within hours of wounding and precedes proliferation [18, 19]. Proliferation starts at day or so later and is needed to supply a sufficient number of keratinocytes to cover the wound surface [17]. Proliferating keratinocytes are located distal to the leading edge of the wound and are found in basal epidermal stem cells, hair follicle bulges, and sebaceous glands [18, 19]. The contribution of keratinocyte proliferation to reepithelialization has been shown through the use of inhibitors. Inhibition of proliferation by 5-fluorouracil (5-FU) or mitomycin-c impedes reepithelialization [19, 20]. In an alternative approach, genetic overexpression of cyclin-dependent kinase inhibitor 1B lowers the rate of epidermal migration, establishing the contribution of proliferation to reepithelialization [20]. Growth factors produced as a result of injury are released by a number of cell types to stimulate keratinocyte proliferation [21], and integrins on the keratinocyte surface facilitate accumulation of intracellular signaling mediators to enhance proliferation [17]. Even after epithelial closure, proliferation of keratinocytes continues [19, 22]. Growth factors that stimulate keratinocyte migration and proliferation are shown in Table 1.

## 3. The Impact of Diabetes on Reepithelialization

Diabetes is a metabolic disease characterized by hyperglycemia and has two major forms, type 1 diabetes mellitus (T1DM) and type 2 diabetes mellitus (T2DM). T1DM results from an absolute insulin secretion deficiency, while T2DM is caused by insulin resistance and inadequate compensatory insulin secretion [23]. Diabetic wounds have a microenvironment with elevated levels of glucose, advanced glycation end products (AGEs), reactive oxygen species (ROS), and inflammatory cytokines. High glucose levels reduce keratinocyte migration and proliferation in vivo and in scratch wound assays *in vitro* [24]. Advanced glycation end products (AGEs) impair keratinocyte proliferation and migration with effects that are similar to those induced by high glucose [25]. Interestingly, the reduced migration is linked to high production of MMP9 and reduced expression of tissue inhibitor of matrix metalloproteinase, TIMP. Oxidative stress is a disturbance in prooxidant and antioxidant balance and is characterized by increased levels of reactive oxygen species (ROS) [26]. Diabetes increases ROS levels that are harmful to wound healing and inhibit keratinocyte migration and proliferation [27]. Furthermore, increased ROS levels stimulate apoptosis, which may be an additional factor that impairs the healing response in diabetes [28]. High levels of ROS induce the production of inflammatory cytokines such as TNF*α*. When TNF is inhibited in diabetic wounds, reepithelialization is significantly enhanced [29]. In addition, diabetes causes prolonged chemokine expression that leads to difficulty in downregulating inflammation, contributing to poor healing outcomes [30]. In diabetic animals, high levels of TNF and reduced levels of TGF-beta are linked to poor healing outcomes that are also characterized by a high percentage of M1 macrophages relative to M2 macrophages at later time points [31]. When TNF is inhibited, there is restoration of M2 macrophage levels and improved healing [31]. Thus, the failure to switch from an M1 to M2 phenotype may interfere with resolution of inflammation and delayed diabetic wound healing.

Diabetic wounds that heal slowly are more susceptible to the formation of a biofilm on the wound surface and the formation of chronic, nonhealing wounds [32]. All dermal and mucosal wounds must cope with the presence of bacteria. Slowly healing wounds are susceptible to biofilm formation at the wound site [33, 34]. Wound infection may impair healing through prolonged inflammation that interferes with the transition from the inflammatory and proliferative phases to the maturation phase of healing [35, 36]. In addition, high levels of ROS contribute to reduced microbial diversity that increases the likelihood that a biofilm-forming specie will dominate the wound, promote biofilm formation, and prevent wound closure [37]. Thus, reversing the redox imbalance at wound sites may one day be an adjunct to limit the formation of chronic wounds [38].

In human dermal wounds, the dominant bacteria are *Staphylococci* (25%), *Corynebacterium* (20%), *Clostridiales* (18%), and *Pseudomonas* (12%) [39]. *Staphylococcus aureus* is particularly linked to delayed reepithelialization and closure [40]. High throughput sequencing studies indicate that strain-level variations of *Staphylococcus aureus* as well as *Corynebacterium striatum* and *Alcaligenes faecalis* correspond with poor wound healing outcomes in diabetic humans [41]. Moreover, due to antibiotic resistance, antibiotics are less effective than debridement in reducing wound biofilm and improving healing in diabetic patients [41]. Bacteria can influence the events of healing by inducing inflammation that retards reepithelialization as described above. Bacteria can also have direct effects on keratinocytes by stimulating apoptosis, reducing migration, and decreasing proliferation [42]. Thus, the presence of bacteria disrupts the careful interplay between keratinocytes and immune cells that is necessary for initial inflammation, subsequent resolution of inflammation, and successful transition to later stages in healing. An effective host response is particularly important in oral wounds as deletion of IL-1 has a deleterious effect on oral mucosal healing that is rescued by antibiotic treatment [43]. In contrast, IL-1 deletion has only a small effect on dermal wounds.

## 4. FOXO1, Diabetes, and Reepithelialization

Transcription factors organize cellular activity to orchestrate a coordinated response to wound healing. The forkhead box O1 (FOXO1) transcription factors stimulate a diverse array of cellular activities including differentiation, apoptosis, DNA repair, response to oxidative stress, inflammation, and the expression of growth factors [44, 45]. There are four different isoforms of FOXO, FOXO1, FOXO3, FOXO4, and FOXO6. For wound healing, this review will focus on FOXO1 specifically, since it is the best studied. Activation of FOXO1 results in its translocation to the nucleus and regulation of gene transcription. FOXO1 expression and activation are significantly increased in keratinocytes by wounding [44, 46, 47]. Keratinocyte-specific FOXO1 deletion results in delayed dermal and mucosal healing [15, 47–49] since FOXO1 activity in keratinocytes is needed for normal keratinocyte migration, reepithelialization and keratinocyte-stimulated connective tissue formation, and angiogenesis in the wound bed [48, 50].

FOXO1 regulates several genes that participate in wound repair. TGF-*β*1 is an important FOXO1 gene target. FOXO1 deletion results in significantly reduced TGF-*β*1 expression in keratinocytes *in vivo* [15, 50]. This regulation is significant since TGF-*β*1 plays a primary role in reepithelialization. TGF-*β* treatment enhances reepithelialization in porcine cutaneous wounds *in vivo* and stimulates human keratinocyte closure of scratch wounds *in vitro* and keratinocyte migration in transwell assays [51, 52]. TGF-*β* application can also accelerate incisional wound closure [14, 53]. TGF-*β* stimulates keratinocyte migration in part, by inducing integrin expression and by recruiting macrophages and fibroblasts to wound areas. TGF-*β*1 rescues impaired reepithelialization caused by FOXO1 deletion, demonstrating that TGF-*β*1 is critical for optimal reepithelialization *in vivo*. FOXO1 induces TGF-*β*1 by binding to the TGF-*β*1 promotor to upregulate its transcriptional activity [15, 50]. FOXO1 also regulates the expression of integrins-*β*6 and -*α*3 needed for keratinocyte migration [8]. Deletion of FOXO1 *in vitro* reduces expression of these integrins, which causes decreased migration that is rescued by integrin overexpression [15]. FOXO1 also protects keratinocytes from oxidative stress by activating antioxidant defense and DNA repair enzymes. Lineage-specific *FOXO1* deletion in keratinocytes *in vivo* increases oxidative damage and in vitro enhances ROS levels, increases oxidative damage, enhances apoptosis, and interferes with keratinocyte migration [15]. The significance of antioxidant expression in keratinocyte migration is shown by rescue of migration in *FOXO1*deleted keratinocytes by application of exogenous antioxidants [15]. In contrast to results above, global haploinsufficiency of (*FOXO1*^+/−^ mice) has been reported to accelerate healing [54]. This may indicate that the FOXO1 expression plays different roles in the healing process in different cell types (global vs. lineage-specific deletion) [15, 54].

**Figure 1:**
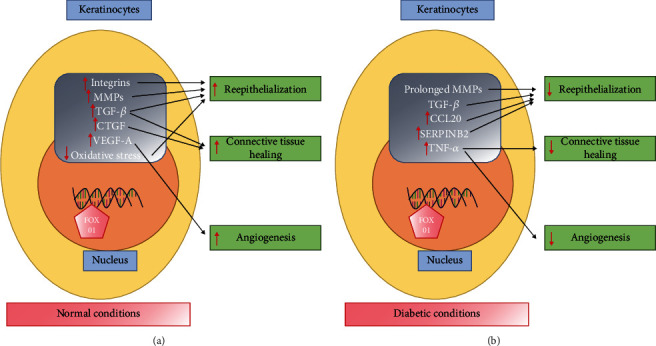
(a) Under normal conditions, FOXO1 promotes reepithelialization through upregulating the expression of integrins, MMPs, TGF-*β*, and antioxidants. FOXO1 promotes connective tissue healing through induction of the TGF-*β* and CTGF expression and stimulates angiogenesis via upregulating VEGF-A. (b) In diabetic conditions, FOXO1 exhibits reduced binding to the TGF-*β*1 promoter to diminish the TGF-*β*1 expression. However, its interaction with a number of factors that inhibit healing when expressed at high levels is increased including MMP-9, CCL20, IL-36*γ*, and SERPINB2 which hamper reepithelialization. FOXO1 is induced by high levels of glucose, advanced glycation end products, and TNF that are elevated in diabetic wounds.

Diabetes impairs reepithelialization by inhibiting keratinocyte migration in both skin and mucosal wounds [47, 50]. There are several mechanisms through which diabetes exerts this effect. As discussed above, diabetes is associated with high levels of glucose, greater formation of advanced glycation end products (AGEs), and enhanced production of inflammatory mediators such as TNF, all of which can inhibit keratinocyte migration [47]. High glucose and AGEs cause molecular changes that diminish the capacity FOXO1 to stimulate TGF-*β*1 expression (Figure 1) [47]. Diabetes also enhances the production of factors that interfere with wound healing when they are produced at high levels (Figure 1). In type 2 diabetic foot ulcers, there is an excessive MMP expression and activity, with increased levels of MMP1, -2, -8, and -9 and reduced levels of TIMP-2 [55]. High levels of MMPs degrade extracellular matrix and limit the ability of keratinocytes to migrate over the wound bed [9]. Interestingly, the negative effect of high glucose on keratinocyte migration is improved by addition of an MMP9 inhibitor or by reducing FOXO1 activity [56]. In type 1 diabetic animals, *FOXO1* ablation *in vivo* reduces high levels of the MMP9 expression [56]. *In vitro* experiments demonstrate that high glucose increases FOXO1 binding to the MMP9 promoter and enhances MMP9 luciferase reporter activity through a FOXO1-dependent mechanism [56]. It is also noteworthy that the absolute level of MMP activity is important, as the absence of MMPs is also problematic. Thus, like many factors, MMP levels need to be tightly regulated at an optimal level for healing to progress since both their absence or overexpression can be damaging to wound healing [57].

FOXO1 in diabetic conditions can enhance the expression of factors that inhibit reepithelialization. In particular, high glucose, AGEs, or inflammation increase FOXO1 binding to the promoters of proinflammatory factors such as CCL20 and IL-36*γ* [50]. High levels of CCL20 and IL-36*γ* interfere with keratinocyte migration and are significantly elevated in keratinocytes in type 1 diabetic mucosal wounds compared to normoglycemic wounds [50]. Inhibition of both CCL20 and IL-36*γ* significantly improve keratinocyte migration when keratinocytes are tested in high glucose or AGE-supplemented media. Like MMP9, the normal expression of IL-36*γ* is likely to be important for reepithelialization as someIL-36*γ* is needed for adequate keratinocyte proliferation in healing wounds [58, 59]. Thus, a minimum level of IL-36*γ* may be required for normal wound healing, while too much IL-36*γ* may be detrimental and contribute to proinflammatory processes as in psoriatic plaques [60] and in response to bacterial infection [61]. Although IL-36*γ* expression has been shown to increase in keratinocytes in diabetic mucosal wounds in vivo and by high glucose and AGES in vitro [50], another study showed that the expression of IL-36*γ* was not elevated in full thickness diabetic wounds from skin biopsies [59]. The reason for the apparent discrepancy is unknown, but it could reflect differences between mucosal and skin wounds.

In summary, diabeteshas several negative effects on keratinocytes that are caused by a change in FOXO1 activity modulated by high glucose or AGEs. Thus, FOXO1 exerts different effects under normal compared to diabetic conditions. On a molecular level, high glucose and AGEs increase FOXO1 binding to the promoter regions of some genes, while reducing FOXO1 interaction with the promoter regions of other genes. For example, high glucose or AGEs diminish FOXO1 binding to the promoter region of TGF-*β*1 and reduce TGF-*β*1 expression. However, high glucose or AGEs increase FOXO1 interaction with the promoters of MMP9, CCL20, and IL-36*γ* to increase their expression to high levels, which interferes with keratinocyte migration [50, 62]. Studies in progress suggest that high glucose and AGEs modify FOXO1-DNA interactions through epigenetic mechanisms that may involve changes in DNA methylation as well as histones.

## 5. Keratinocytes, Connective Tissue Healing, Diabetes, and FOXO1

The crosstalk between epidermal keratinocytes and dermal fibroblasts is needed for normal connective tissue healing [63]. Keratinocytes stimulate fibroblasts and myofibroblasts through production of growth factors such as TGF-*β* and CTGF (Table 1) [49]. Fibroblasts play a critical role in connective tissue healing by producing and remodeling extracellular matrix [64]. Myofibroblasts, a subset of fibroblasts, are responsible for connective-tissue compaction and wound contraction [65]. *In vitro* experiments show that TGF-*β*1 production and activation are upregulated in keratinocytes to stimulate fibroblasts in cocultures [66]. Interestingly, keratinocyte-specific deletion of FOXO1 reduces keratinocyte-produced TGF-*β*1 and impairs connective tissue healing, demonstrating the importance of keratinocytes in the wound healing process [15]. Thus, without FOXO1 produced by keratinocytes, there is diminished numbers of fibroblasts and myofibroblasts caused by reduced proliferation of these cells [49]. Mechanistically, this is tied to FOXO1-induced TGF-*β*1 as defective connective tissue matrix formation in mice with keratinocyte-specific FOXO1 deletion is rescued by addition of exogenous TGF-*β*1 [15]. However, addition of TGF-*β*1 has little effect on connective tissue healing of wounds in control mice, showing that under normal conditions, production of TGF-*β*1 is sufficient and that the addition of more TGF-*β*1 is not helpful.

TGF-*β* can stimulate connective tissue formation by induction of connective tissue growth factor (CTGF), also known as cellular communication network factor-2 (CCN-2) [49, 68]. Application of CTGF *in vivo* stimulates fibroblast proliferation and deposition of collagen [69]. Furthermore, CTGF inhibition reduces the quantity and quality of granulation tissue [69]. In addition to TGF-*β*1, keratinocytes secrete TGF-*β*2. *In vivo* experiments show that TGF-*β*2 induces recruitment of fibroblasts to the wound site and increases collagen deposition and scar formation *in vivo* [70]. Thus, TGF-*β* produced by keratinocytes can induce CTGF in keratinocytes and in cells in the connective tissue of healing wounds [49, 71]. *FOXO1* ablation reduces keratinocyte-produced TGF-*β*1, which leads to a reduction in the CTGF expression [49] and less vigorous production of connective tissue matrix in diabetic wounds [49]. In contrast, the excessive TGF-*β*1 expression may be a key factor in wound fibrosis [67].

## 6. Keratinocytes, Angiogenesis, and FOXO1

Angiogenesis is essential in wound healing and is triggered by hypoxia that results from vascular disruption in wounded tissue [72]. Vascular endothelial growth factor-A (VEGF-A) is the primary isoform of VEGF in wounds and stimulates endothelial cells to proliferate and form vessels. Keratinocytes promote angiogenesis in mucosal and skin wounds through secretion of growth factors [48, 73]. Studies in human wounds and animal models demonstrate that VEGF is produced by keratinocytes during the healing process. Mice with lineage-specific VEGF-A deletion in keratinocytes have delayed wound closure and reduced angiogenesis [74]. In addition to VEGF-A, keratinocytes produce the proangiogenic factor, mitogen-regulated protein 3 (MRP3) [73], and TGF-*β*1, which are needed for normal angiogenesis [75].

Hypoxia-inducible factor-1 (HIF-1) is a transcription factor that stimulates the VEGF-A expression [72]. FOXO1 also participates in VEGF-A transcriptional regulation and stimulates wound angiogenesis by stimulating the VEGF-A expression [48]. *FOXO1* deletion reduces VEGF-A protein levels in wounded mucosal epithelium *in vivo* [48], which results in reduced vascular density and a diminished number of proliferating endothelial cells *in vivo* at wound sites [48]. Interestingly, FOXO1 in chondrocytes also controls VEGF-A expression and angiogenesis in long bone fracture healing [76]. FOXO1 directly interacts with the *VEGF-A* promoter thereby inducing *VEGF-A* promoter activity and VEGF-A expression [48]. A large animal model further supports the role of FOXO1 in promoting angiogenesis as shown by reduced neovascularization when a FOXO1-specific inhibitor is applied [48]. Global deletion of *FOXO1* causes embryonic lethality due to vascular failure [77], and *FOXO1*-deficient endothelial cells show impaired angiogenesis in vivo [78].

Diabetes negatively affects wound healing by interfering with angiogenesis [72]. Diabetic human and animal wounds with reduced angiogenesis have diminished vascularity and capillary density. Defective VEGF production has been reported in keratinocytes from diabetic db/db mice compared with keratinocytes from normal mice [79]. Reduced M2 macrophage levels in diabetic wounds may also contribute to impaired angiogenesis [72]. In addition to having reduced expression of proangiogenic factors, diabetic wounds also have increased antiangiogenic factors produced by keratinocytes [80, 81]. Thrombospondin-1(TSP-1) is an angiogenesis inhibitor [82] and produced in greater amounts by keratinocytes in high-glucose environment [83]. Thus, it is possible that an increase in TSP-1 acts as a brake in the formation of new blood vessels in diabetic wounds. FOXO1 may contribute to diabetes-impaired angiogenesis [84]. FOXO1 enhances apoptosis of microvascular endothelial cells in diabetic animals *in vivo* and induces apoptosis in microvascular cell endothelial cells and pericytes exposed to high glucose *in vitro* [85]. The increased apoptosis may negatively affect angiogenesis. In high glucose media, FOXO1 disrupts human microvascular endothelial cell formation of vascular tubes [86]. Local injection of adenovirus-expressing FOXO1 in type 2 diabetic mice with skin wounds reduces vascular density [87]. Thus, in a high glucose, diabetic environment FOXO1 may affect endothelial cells to disrupt angiogenesis and impair wound healing.

## 7. Summary

Wound healing is a complex process with many cells and factors involved. Keratinocytes play crucial roles in this process by reepithelialization of open wounds and by the production of factors that influence connective tissue healing and angiogenesis (Table 1). FOXO1 is essential in upregulating the wound healing activity in keratinocytes by stimulating expression of factors that promote healing including TGF-*β*1, integrins, and antioxidants. In diabetic wounds, FOXO1 activity changes to take on a negative role. This is due to the influence of conditions present in the diabetic wound such as high levels of glucose, increased AGEs, and increased TNF levels. These factors decrease the interaction of FOXO1 with the TGF-*β*1 promoter and have a detrimental effect on both reepithelialization and formation of new connective tissue. Furthermore, diabetic conditions increase FOXO1 interactions with the promoters of other genes to enhance their expression. This has a negative effect since high levels of MMP9, CCL20, and IL-36*γ* impede keratinocyte migration and interfere with reepithelialization.

## Figures and Tables

**Table 1:** Examples of growth factors produced by keratinocytes that promote wound healing.

Growth factor	Function	*In vivo* experiments and results	*In vitro* experiments and results
FGF2	Reepithelialization and granulation tissue formation	Global deletion of FGF2 in mice cause delayed skin wound healing [16].	FGF2 application stimulates human keratinocytes migration *in vitro* [88].
TGF-*β*	Inflammation	Global deletion of TGF-*β* in mice cause delayed wound healing [12]; TGF-*β* application in rats at the wound site accelerates wound healing [14, 53].	TGF-*β* application stimulates keratinocyte migration in skin explants by upregulating the expression of integrins [51, 52].
	Reepithelialization		
	Keratinocytes migration		
	Granulation tissue formation		
VEGF-A	Angiogenesis	Keratinocyte-specific deletion of VEGF-A in mice reduces blood vessel formation at the wound site [74]. Topical VEGF in diabetic mice accelerate wound healing [74].	VEGF-A stimulates endothelial cells *in vitro* to increase tube formation [74].
HB-EGF	Reepithelialization	Keratinocyte-specific HB-EGF-deficient mice have delayed migration and wound closure [89].	HB-EGF overexpression by epidermal keratinocytes increases motility [90].
